# Enhancement of intestinal barrier function and alleviation of mycophenolic acid toxicity by a probiotic-conditioned medium *in vitro*

**DOI:** 10.3389/fnut.2026.1809197

**Published:** 2026-04-14

**Authors:** Tiziana Di Renzo, Annamaria Di Giacomo, Anna Reale, Daniela Iovanna, Stefania Moccia, Carmela Spagnuolo, Mauro Cataldi

**Affiliations:** 1 Institute of Food Sciences, National Research Council, Avellino, Italy; 2 Section of Pharmacology, Department of Neuroscience, Reproductive Sciences and Dentistry, Federico II University of Naples, Naples, Italy

**Keywords:** immunosuppressant drugs, mycophenolate mofetil, mycophenolic acid, pharmacomicrobiomic, probiotics

## Abstract

Immunosuppressant drugs may damage the intestinal barrier (IB) either indirectly by promoting dysbiosis, or directly by cytotoxic effects exerted on intestinal cells. This has been demonstrated, for instance, with the inosine monophosphate inhibitor mycophenolic acid (MPA) and with its ester prodrug mycophenolate mofetil (MMF). These two drugs can cause severe gastrointestinal toxicity in transplant patients receiving them for rejection prophylaxis. We investigated whether a multistrain probiotic preparation could protect against MPA- and MMF-induced IB damage using differentiated CaCo-2 cells as *in vitro* model. We obtained an acellular probiotic conditioned medium by culturing the multistrain probiotic preparation in DMEM + FBS, either with or without MPA or MMF. The probiotic conditioned medium reduced IB permeability when added to the upper compartment of the transwell both in the presence and in the absence of these immunosuppressant drugs, as indicated by an increase in transepithelial electrical resistance. The probiotic conditioned medium also prevented cytotoxicity induced by both MPA and MMF and increased the expression of the tight junction proteins zonulin-1 and claudin-5, both of which contribute to barrier tightness. The protective effects of the probiotic medium may depend on preventing free radical damage, as we found that it reduced free oxygen radicals in cells exposed to either MPA, MMF or to the pro-oxidant compound tert-butyl hydroperoxide (tBHP). Finally, the probiotic conditioned medium reduced the activity of the ABC1B1 pump, though this did not result in changes in transwell IB crossing by MPA or MMF. In conclusion, we demonstrated that a multistrain probiotic preparation can protect the intestinal barrier from toxicity induced by MPA and MMF. The fact that this was a postbiotic effect, as it occurred in the absence of probiotic microorganisms, could be relevant for immunocompromised patients, as probiotic bacteria could potentially induce opportunistic infections in these subjects.

## 1 Introduction

The intestinal barrier (IB) represents a highly dynamic and selective interface that separates the host from the intestinal lumen while allowing controlled exchange of nutrients, metabolites, and immune signals. It consists of multiple, functionally integrated components: the mucus layer secreted by goblet cells, the epithelial monolayer sealed by intercellular junctions, the lamina propria immune system, and the commensal microbiota that plays a crucial role in maintaining homeostasis, through multiple mechanisms including the production of short-chain fatty acids (SCFAs) such as butyrate, propionate, and acetate, as well as of immunomodulatory polysaccharides like Polysaccharide A (PSA) from *Bacteroides fragilis* (1). This complex architecture ensures both physical protection and immunological tolerance toward luminal antigens and microorganisms (2, 3).

Anatomical and functional damage to the IB contributes to the pathophysiology of multiple clinical conditions, one particularly relevant example being the post-transplantation state (4–6). Indeed, various IB-damaging factors coexist and may interact synergistically in transplant recipients. The most significant of these are gut dysbiosis and pharmacological treatment with immunosuppressant drugs for rejection prophylaxis. Gut dysbiosis is defined as a reduction in the diversity of intestinal microbiota, overgrowth of pathobionts and loss of beneficial bacteria, that in turn lead to barrier integrity impairments (7, 8).

This disruption to the intestinal barrier occurs through multiple mechanisms, including the production of toxic substances that damage the intestinal epithelium directly, a decrease in SCFAs that reduces the expression of tight junction (TJ) proteins, a reduction in mucus production, impaired epithelial regeneration and inflammation that activates toll receptors and releases proinflammatory cytokines (9, 10). Consequently, gut dysbiosis promotes microbial translocation, systemic inflammation and systemic complications (1, 10, 11). For example, a large metagenomic study of liver and kidney transplant recipients found that persistent dysbiosis was linked to higher post-transplant mortality (12). Furthermore, alterations in the gut microbiome have been associated with antibody-mediated rejection in kidney transplant recipients, indicating a potential mechanistic role of the microbiome in alloimmunity (13). Immunosuppressant drugs may damage the intestinal barrier not only by promoting dysbiosis but also through direct toxicity. In fact, experiments in the CaCo-2 cell transwell model of the intestinal barrier have demonstrated that exposure to tacrolimus or cyclosporine reduces the expression of zonulin-1 (ZO-1) and increases epithelial permeability (14). Similarly, the immunosuppressant drug mycophenolic acid -an inosine dehydrogenase inhibitor which is also available as the prodrug ester mycophenolate mofetil- damages the intestinal barrier by reducing the expression of TJ proteins through enhanced oxygen free radical generation (15, 16).

Though their efficacy is still uncertain (17), probiotics defined as live microorganisms that, when administered in adequate amounts, confer demonstrable health benefits to the host (18–21) are frequently given to transplant patients to reduce the detrimental effects of intestinal dysbiosis. These beneficial effects may be partly explained by a reduction in structural and functional damage to the intestinal barrier (22). Indeed, *Lacticaseibacillus rhamnosus* and *Lactoplantibacillus plantarum* have been shown to improve tight junction integrity, as assessed by transepithelial electrical resistance (TEER) and ZO-1 localization, in CaCo-2 cells (23). They have also been shown to confer protection in a rat model of gut injury (24). Similarly, multispecies probiotic formulation reduced the increase in intestinal permeability induced *in vitro* and *in vivo* by incubation with conditioned medium from colonic biopsies or fecal supernatant from patients with inflammatory bowel disease (23). Furthermore, in 6-week-old mice with 5/6 nephrectomy, orally administered lactobacilli reduced gut mucosa leakage by increasing adhesion molecule expression (25). In a 0.2% adenine-induced chronic kidney disease (CKD) mouse model, *Lacticaseibacillus paracasei* and *Lactiplantibacillus plantarum* reversed gut dysbiosis, reducing renal fibrosis and abnormal intestinal barrier permeability (26). Likewise, *Lactiplantibacillus plantarum* administration modulated genes involved in cell adhesion and tight junction maintenance in the small intestine of human volunteers (27). A recent experimental study performed on mice showed that a commercial probiotic preparation containing *Bifidobacterium longum*, *Lactobacillus acidophilus*, and *Enterococcus faecalis* improved MPA-induced colitis and increased the expression of ZO-1 and occludin compared to animals that received MPA only (28). The authors also demonstrated that probiotic treatment prevented MPA-induced intestinal dysbiosis, suggesting that this may be the key mechanism underlying the protective effect against drug-induced intestinal damage. In the present paper, we therefore studied the effect of an acellular preparation, which is made by culturing probiotic microorganisms in a cell culture medium and then removing the bacteria, on intestinal barrier function and MPA/MMF-induced cytotoxicity in an *in vitro* model. Our experiments focused on early time points to evaluate whether PP can precondition intestinal epithelial cells and mitigate the initial molecular disturbances, thereby enhancing barrier resilience before overt damage occurs.

## 2 Materials and methods

### 2.1 Chemicals

Mycophenolate mofetil (MMF) and mycophenolic acid (MPA), produced by the Cayman Chemical Company (Ann Arbor, MI, United States), were obtained from Cabru S.A.S. (Arcore, Milan, Italy). The purity of MMF and MPA was ≥95% and ≥98%, respectively. The drugs were dissolved in dimethyl sulfoxide (DMSO) to obtain 5 mM stock solutions, which were then aliquoted and stored at −20 °C. The HPLC-grade acetonitrile was obtained from Carlo Erba (Cornaredo, MI, Italy); DMSO, hydrochlorothiazide, and orthophosphoric acid were obtained from Sigma-Aldrich (Milan, Italy). All reagents and solvents were of analytical grade.

### 2.2 Experimental design

A conditioned medium was prepared by culturing probiotic bacteria from a commercial multistrain preparation in CaCo-2 cell culture medium. This medium was then used to evaluate the probiotic effect on damage to the intestinal barrier induced by MPA or MMF. For this purpose, a transwell model was employed. Briefly, CaCo-2 cells (a cell line derived from human colorectal carcinoma that spontaneously differentiates into enterocytes when confluent *in vitro*) were grown on the membrane of transwell inserts until confluence was reached. Then, the culture medium in the upper compartment of the transwell insert (representing the intestinal lumen in this model) was replaced with conditioned medium containing MPA, MMF, or the respective vehicle. In controls, these substances were dissolved in normal cell culture medium. After a 6 h incubation period, the effect of these treatments on barrier permeability was assessed by measuring TEER. To characterize the toxicity of MPA and MMF and the potential protective effect of the conditioned medium mechanistically, we measured cell viability and the expression of the intercellular adhesion proteins zonulin-1 (ZO-1) and claudin-5 in CaCo-2 cells cultured in plastic dishes until they reached confluence and acquired a fully differentiated status. We also evaluated the activity of the plasma membrane pump ABCB1, a detoxifying mechanism that can extrude drugs from the cytoplasm, by measuring the intracellular accumulation of its fluorescent substrate, rhodamine 123 (Rho-123). We determined the impact on drug absorption again using the transwell model by measuring the concentration of MMF and MPA at the beginning and end of a 6 h incubation period in both the upper and lower compartments using RP-HPLC.

### 2.3 Preparation of probiotic conditioned medium

A commercially available probiotic dietary supplement in capsule form was used in this study. Each capsule contained the following microbial strains: *Lactiplantibacillus plantarum* (5 × 10^9^ cells/capsule), *Lacticaseibacillus rhamnosus* (7.5 × 10^9^ cells/capsule), *Lactobacillus acidophilus* (4.5 × 10^9^ cells/capsule), *Lacticaseibacillus casei* (3 × 10^9^ cells/capsule), *Streptococcus thermophilus* (1.5 × 10^10^ cells/capsule), and *Bifidobacterium lactis* (1.5 × 10^10^ cells/capsule).

For the preparation of the conditioned medium, the capsule content was resuspended in Dulbecco’s Modified Eagle Medium (DMEM; GIBCO, Thermo Fisher Scientific) supplemented with 10% fetal bovine serum (FBS; GIBCO, Thermo Fisher Scientific), in presence of either 50 μM MMF, 50 μM MPA, or their vehicle [dimethyl sulfoxide (DMSO)].

The bacterial suspension was adjusted to a final concentration of approximately 7 log CFU/mL and incubated at 37 °C for up to 6 h under anaerobic conditions (Supplementary Figures 1, 2). This concentration was selected to reflect the estimated number of viable probiotic cells that reach the intestinal epithelial surface after gastrointestinal transit, considering the reduction in viability that occurs during passage through the gastrointestinal tract, as reported in previous studies (29, 30).

After 3 and 6 h of incubation, time points chosen to reflect realistic exposure periods within the intestinal environment, the suspension was centrifuged (6,000 rpm for 15 min) and the supernatant was collected and sterile-filtered through a 0.22 μm membrane filter (Sarstedt) to obtain cell-free probiotic-conditioned medium (PP medium). The conditioned medium was stored at −20 °C until use in experiments involving differentiated CaCo-2 cells.

### 2.4 CaCo-2 cell culture and transwell preparation

The CaCo-2 cells were obtained from ATCC (ATCC^®^ HTB-37™) and cultured in 100-mm Petri dishes in DMEM (Dulbecco’s Modified Eagle Medium) supplemented with 1% L-glutamine (200 mM), 5,000 IU/mL penicillin, 5,000 μg/mL streptomycin (Thermo Fisher Scientific), 1% non-essential amino acids, and 10% FBS (GIBCO, Thermo Fisher Scientific). The cells were incubated at 37 °C in a humidified atmosphere containing 5% CO_2_, and the medium was replaced every 48 h. To recreate an intestinal barrier *in vitro*, the cells were seeded onto polyethylene terephthalate (PET) filter inserts (Sartorius, 0.4 μm pore size), which were placed in 12-well cell culture plates. The cells were cultured in complete medium for 21 days until they reached confluence and full enterocyte differentiation. At this point, the cells were ready for experiments involving PP. During this time, the cell culture medium was changed three times a week.

### 2.5 Measurement of TEER (transepithelial electric resistance) measurement

We measured the electrical resistance across the epithelial barrier formed by differentiated CaCo-2 cells on the PET filter of the transwell using a Millicell^®^ ERS 3.0 Digital Voltohmmeter (ERS 3.0; Merck Millipore), according to the manufacturer’s instructions. This resistance is inversely related to barrier permeability and depends primarily on the integrity of the tight junctions. Briefly, the electrodes were sterilized and preconditioned in culture medium. Then, the blank resistance was calculated by placing the two electrodes in the upper and lower compartments of a transwell containing culture medium, but no cells. Next, the electrodes were moved to wells containing CaCo-2 cells. TEER values were obtained after correcting for blank resistance and are expressed as Ω cm^2^ based on the following equation: TEER = (R−R_b) × A, where R is the resistance of the filter insert with cells, R_b is the resistance of the filter alone, and A is the growth area of the filter. The results are expressed as a percentage relative to untreated cells.

### 2.6 Cellular viability assay

Cell viability was determined using the CyQuant Cell Proliferation Assay Kit (ThermoFisher Scientific/Life Technologies), which estimates the number of cells in an *in vitro* culture by measuring the light emitted by a DNA-binding fluorescent probe. The assay was performed as described by Russo et al. (31). Briefly, CaCo-2 cells were grown in Petri dishes until they reached full confluence and enterocyte differentiation. Then, the culture medium was replaced with either normal medium or PP, both of which were supplemented with MMF, MPA, or their vehicle, and renewed every 24 h. After a 72 h incubation period, the CyQuant Direct Reagent was added to the culture media. This reagent contains a DNA-binding fluorescent dye and a background suppressor that blocks staining in dead or damaged cells. After an additional 1-h incubation at 37 °C, fluorescence was measured with a microplate multiwell reader using an excitation wavelength of 485 nm and an emission wavelength of 530 nm. The data were expressed as a percentage of the fluorescence values measured in the control wells.

### 2.7 Immunoblotting

The effect on TJ protein expression of MPA, MMF or their vehicle dissolved either in normal cell culture medium or in PP was investigated by Western blotting in differentiated CaCo-2 cells. After a 6-h incubation with the test compounds, the cells were detached, collected, washed with PBS, and resuspended in a lysis buffer containing 150 mM NaCl, 50 mM Tris/HCl, 1% NP-40, 10 mM EDTA, 10% glycerol, 0.5 mM DTT, 1% protease and phosphatase inhibitor cocktail, and 1 mM PMSF (Merck/Sigma-Aldrich). The protein concentration was determined using a DC protein assay (Bio-Rad), and the samples were prepared by adding 2X Laemmli sample buffer (Bio-Rad). Homogenates were loaded onto 10%–12% SDS-PAGE gels, and electrophoresis was performed in Tris-Glycine buffer. The gels were transferred onto PVDF membranes (Bio-Rad Laboratories, Milan, Italy) using a Trans-Blot Turbo System (Bio-Rad). The membranes were blocked with 5% non-fat dry milk in T-TBS 0.1%, followed by incubation with primary antibodies: Zonulin-1, Claudin-5, Claudin-1, Occludin, α-Tubulin (Cell Signaling), and ABC1B1 (Invitrogen). Immunoblotting was performed using standard procedures and developed with the ECL Plus Western blot detection system (Cytiva, Euroclone, Milan, Italy) with the Chemidoc system (Bio-Rad). Densitometric analysis of the bands was performed using Image Lab software (Bio-Rad).

### 2.8 Measurement of intracellular reactive oxygen species

Reactive oxygen species (ROS) were measured using chloromethyl-2′,7′-dichlorofluorescein diacetate (CM-H_2_DCF-DA), which is a cell-permeable substance that is deesterified to 2,7-dichlorodihydrofluorescein (DCFH) once inside the cell. In the presence of ROS, DCFH is oxidized to the fluorescent compound dichlorofluorescein (DCF). In brief, confluent, differentiated CaCo-2 cells grown in multiwell plastic dishes were exposed to MPA, MMF or their vehicle dissolved either in normal cell culture medium or in PP for 2 h; or cells were stimulated with PP for 2 h and then, the oxidant and free radical-generating compound tert-butyl hydroperoxide (tBHP; 300 μM) was added, after which the cells were incubated for 30 min at 37 °C in a humidified atmosphere containing 5% CO_2_. After this incubation period, a solution of CM-H_2_DCF-DA (10 μM) was added to the extracellular medium (Invitrogen Life Technologies) and the cells were incubated for an additional 30 min. The cells were then washed twice with PBS, after which the fluorescence emitted at 530 nm following excitation at 485 nm was measured using a spectrofluorimeter. Fluorescence intensities were quantitatively normalized to the untreated control condition, which was designated as 100%, and all experimental readings were subsequently expressed as relative percentage values.

### 2.9 Rhodamine 123 accumulation assay

We determined the activity of the ABC1B1 (P-glycoprotein, Pgp) pump by measuring the intracellular accumulation of its fluorescent substrate, Rho-123. Since the pump extrudes this cell-permeable compound, the intracellular accumulation of Rho-123 and the fluorescence that it emits at 485 nm when excited at 535 nm are inversely related to the activity of the pump. Briefly, differentiated CaCo-2 cells treated for 24 h with MPA, MMF or their vehicle, either in PP or normal medium, were incubated at 37 °C with 5 μM Rho-123 for 1 h. After washing with phosphate-buffered saline, the cells were lysed in distilled water. Intracellular levels of Rho-123 were quantified by measuring fluorescence with a microplate reader (Synergy HT, BioTek, Milan, Italy) at excitation and emission wavelengths of 485 and 535 nm, respectively. The results were then normalized per mg of protein content in lysed cells.

### 2.10 Determination of MMF and MPA in bacterial and CaCo-2 cell culture media

The concentrations of MPA and MMF in bacterial and CaCo-2 cell culture media samples were measured by RP-HPLC using the method published by Bolon et al. (32), with minor modifications. Briefly, HPLC separation was performed at 30 °C using a Shimadzu Prominence apparatus fitted with a C18 reverse-phase HPLC column (Zorbax, Omnisphere 5 C18, 250 × 4.6 mm, Agilent Technologies, Netherlands), a precolumn (5 μm C18, 12.5 × 4.6 mm, Agilent Technologies, Netherlands), a photodiode array UV detector, and a column oven. A linear gradient was generated using two mobile phases: phase A consisting of 40 mM H_3_PO_4_ and phase B consisting of 55% phase A and 45% acetonitrile. Specifically, the percentage of phase B was kept at 55% from minute 0 to minute 4, increased to 100% over 13 min (from minute 4 to minute 17), and lowered back to 55% over 3 min (from minute 17 to minute 20). For HPLC analysis, samples of the culture media were extracted with three volumes of an acetonitrile-methanol mixture and dried in a Savant speed-vac concentrator. Prior to HPLC analysis, the evaporated culture samples were resuspended in 100 μL of solution B and centrifuged at 13,000 rpm for 10 min. Fifty microliters of the resuspended samples were injected into the HPLC system using the autosampler injector, and UV light absorption was measured at 254 nm.

### 2.11 Statistical analysis

The data were analyzed using a one-way ANOVA followed by Tukey’s multiple comparison test (GraphPad Prism version 9.5.1). Cellular assays differences between two groups were analyzed using Student’s *t*-test (ExcelMS software). All results are expressed as mean values ± standard deviation (SD). A confidence level of 95% was applied, and differences were considered statistically significant at *p* < 0.05.

## 3 Results

### 3.1 PP enhances intestinal barrier function

**FIGURE 1:**
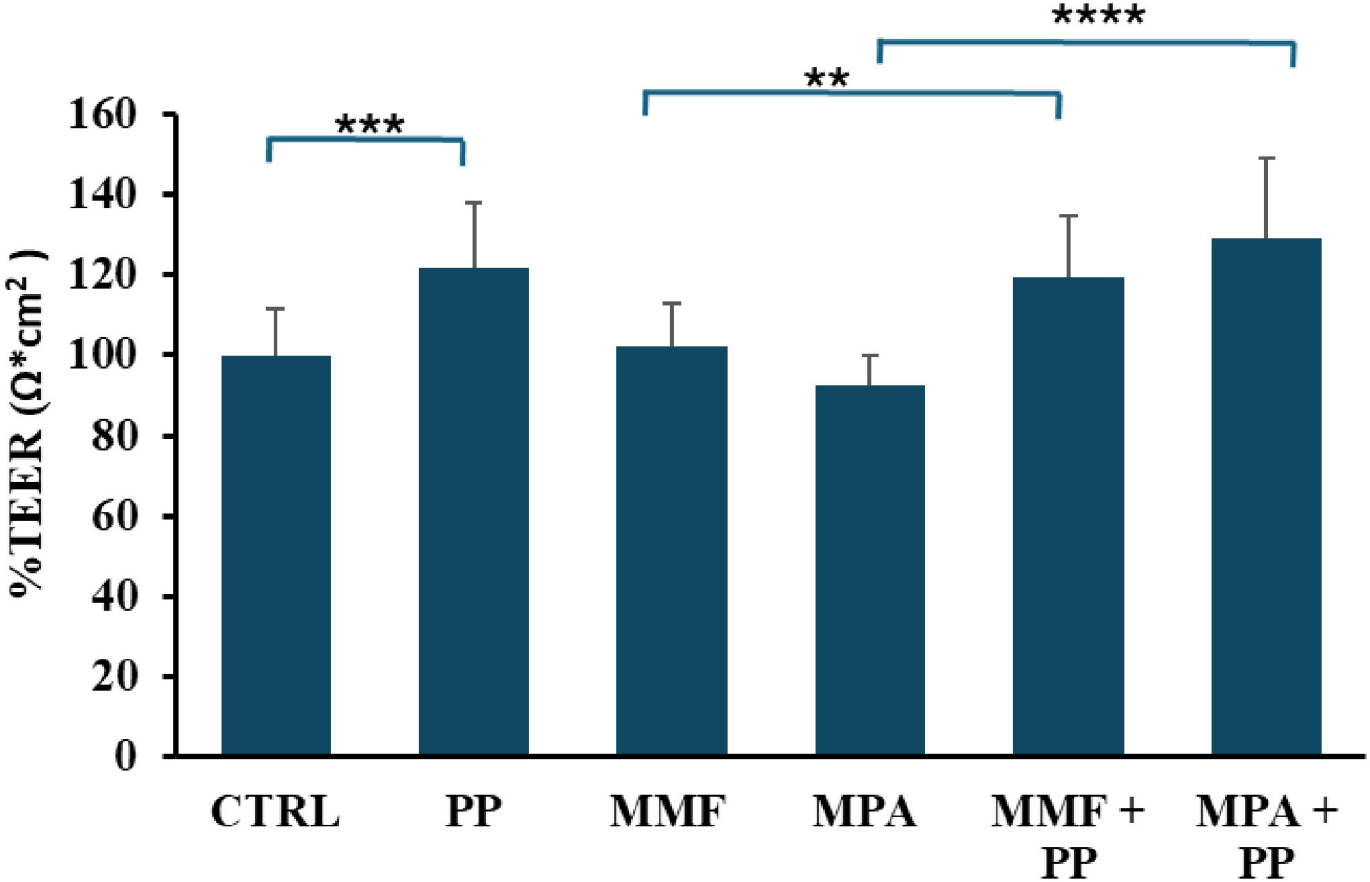
Effect of probiotic-conditioned (PP), Mycophenolic acid (MPA), and mycophenolate mofetil (MMF) on transepithelial electrical resistance (TEER) of differentiated CaCo-2 cells. The bar graph shows the mean and standard deviation (SD) of values measured in six experiments performed with differentiated CaCo-2 cells exposed to either MMF or MPA (both at a concentration of 50 μM) or their vehicle in normal cell culture medium or PP for 6 h. Data are expressed as a percentage of the control group. Asterisks indicate statistical significance after the ANOVA (multiple comparison) analysis: **p < 0.005; ***p < 0.001; ****p < 0.0001.

The effects of PP and the immunosuppressant drugs MPA and MMF on intestinal barrier permeability were assessed by measuring transepithelial electrical resistance (TEER) across filter membranes of transwells onto which CaCo-2 cells had grown until confluence and differentiation. MPA (50 μM), MMF (50 μM), or their vehicle were dissolved in either PP or normal cell culture medium and placed in the apical compartment of the transwell. After a 6 h incubation period, TEER already was significantly higher in transwells exposed to both PP and drugs dissolved in PP respect to controls (Figure 1), indicating that the probiotic preparation ameliorates the barrier conditions. Instead, incubation with MMF and MPA after 6 h did not modify TEER compared with control.

### 3.2 PP increases the expression of tight junction proteins

**FIGURE 2:**
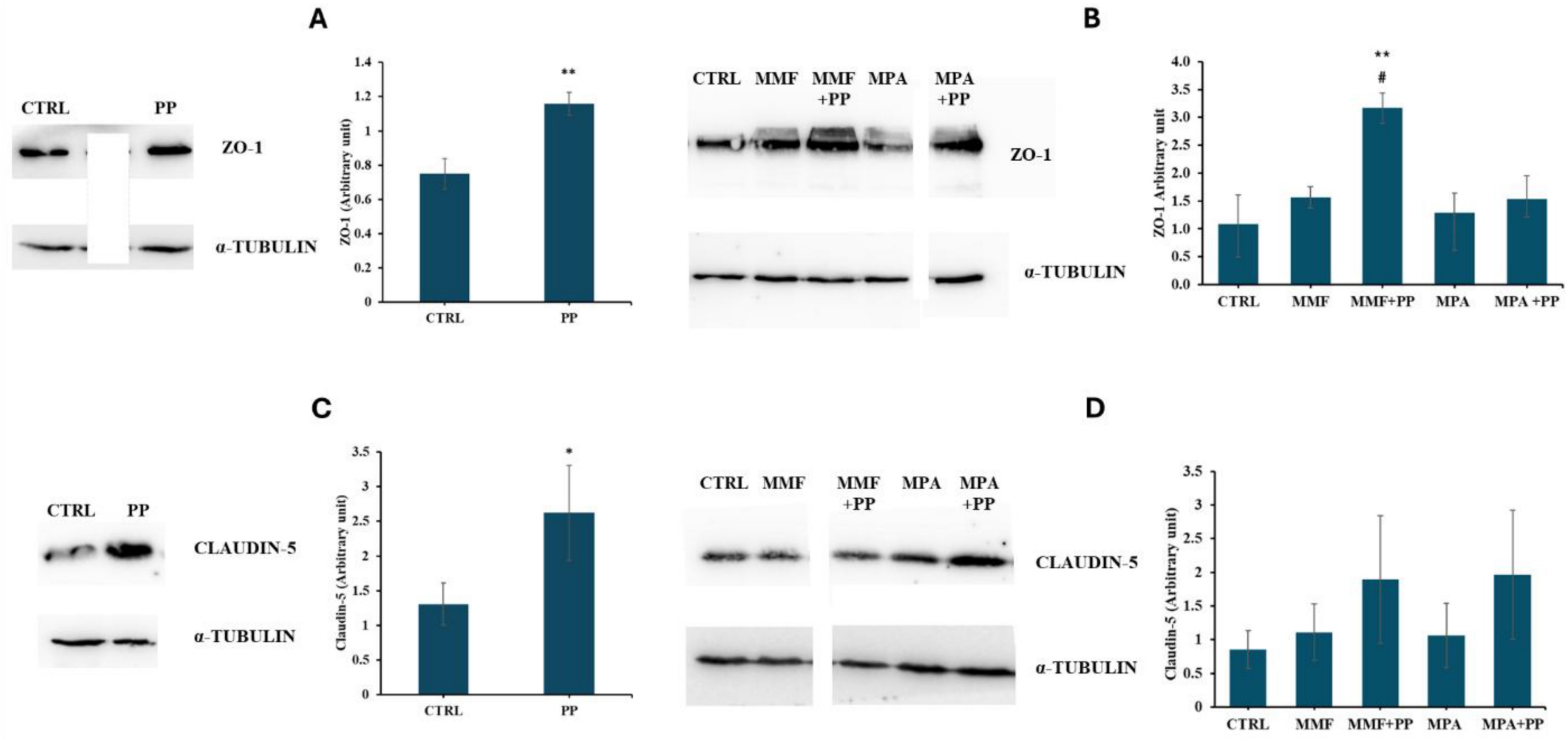
Effect of probiotic-conditioned (PP), Mycophenolic acid (MPA), and mycophenolate mofetil (MMF) on the expression of tight junction proteins. The immunoblots show the expression levels of ZO-1 **(A,B)** and claudin-5 **(C,D)** proteins in lysates of differentiated CaCo-2 cells that were exposed for 6 h to MMF or MPA (both at a concentration of 50 μM) or their vehicle, either in normal cell culture medium or PP. The bar graph shows the mean and standard deviation (SD) of the values of densitometric analysis (arbitrary unit) obtained in four experiments; symbols indicate statistical significance student’s *t*-test (two-group of data) analysis: *p < 0.05, **p < 0.005 compared to untreated cells (CTRL), #*p* < 0.005 compared to MMF.

We then investigated the effect of PP on the expression of tight junction (TJ) proteins as these proteins are the primary determinants of intestinal barrier integrity and their expression has been shown to decrease in CaCo-2 cells exposed to MPA (16), which could also contribute to MPA-induced cell death (33). Western blot experiments (Figure 2) revealed that, after a 6 h incubation period, the expression of claudin-5 and zonulin-1 was significantly higher in CaCo-2 cells exposed to PP than in control cells. Remarkably, the expression-enhancing effect of PP was also observed in the presence of drugs. Thus, evaluating claudin-5 and ZO-1, that are generally modulated rapidly, at early time points allows us to capture the initial protective and barrier-enhancing effects of PP. Instead, claudin-1 and occludin remain unchanged after 6 h of treatment (Supplementary Figure 3), this probably because both proteins are relatively stable TJ components whose expression does not necessarily change within short time frames unless epithelial injury is severe or prolonged (34, 35).

### 3.3 PP prevents MPA-induced cytotoxicity

**FIGURE 3:**
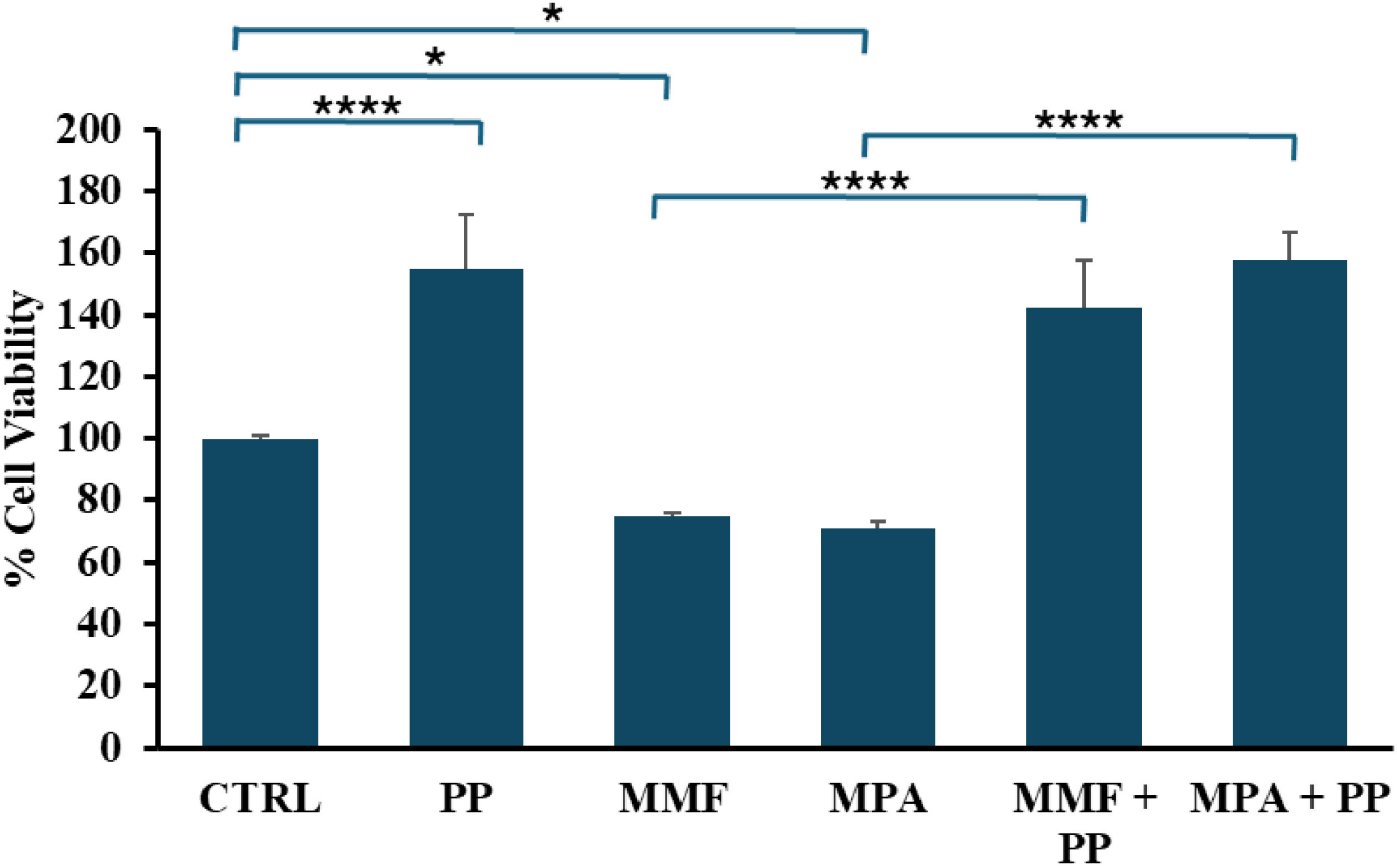
Effect of probiotic-conditioned (PP), mycophenolic acid (MPA), and mycophenolate mofetil (MMF) on cell viability in differentiated CaCo-2 cells. The cells were exposed to either MMF or MPA (both at a concentration of 50 μM) or their vehicle in normal medium or in PP for 72 h prior to measuring CyQuant fluorescence. The bar graph shows the mean and standard deviation (SD) of the values obtained in three experiments, expressed as a percentage of the control. Asterisks indicate statistical significance after the ANOVA (multiple comparison) analysis: *p < 0.05; ****p < 0.0001.

To further investigate the ameliorating effect of PP on intestinal barrier we evaluated its impact on cell viability of differentiated CaCo-2 cells measured with the CyQuant assay after longer exposure. As shown in Figure 3, after 48 h of treatment both MPA and MMF significantly reduced cell viability as compared with controls exposed to normal culture medium only. Conversely, an increase in cell viability above control levels was observed in cells exposed to PP with or without MPA or MMF. These results suggest that PP in CaCo-2 cells exposed to MPA and MMF is able to prevent the cytotoxicity of these drugs.

**FIGURE 4:**
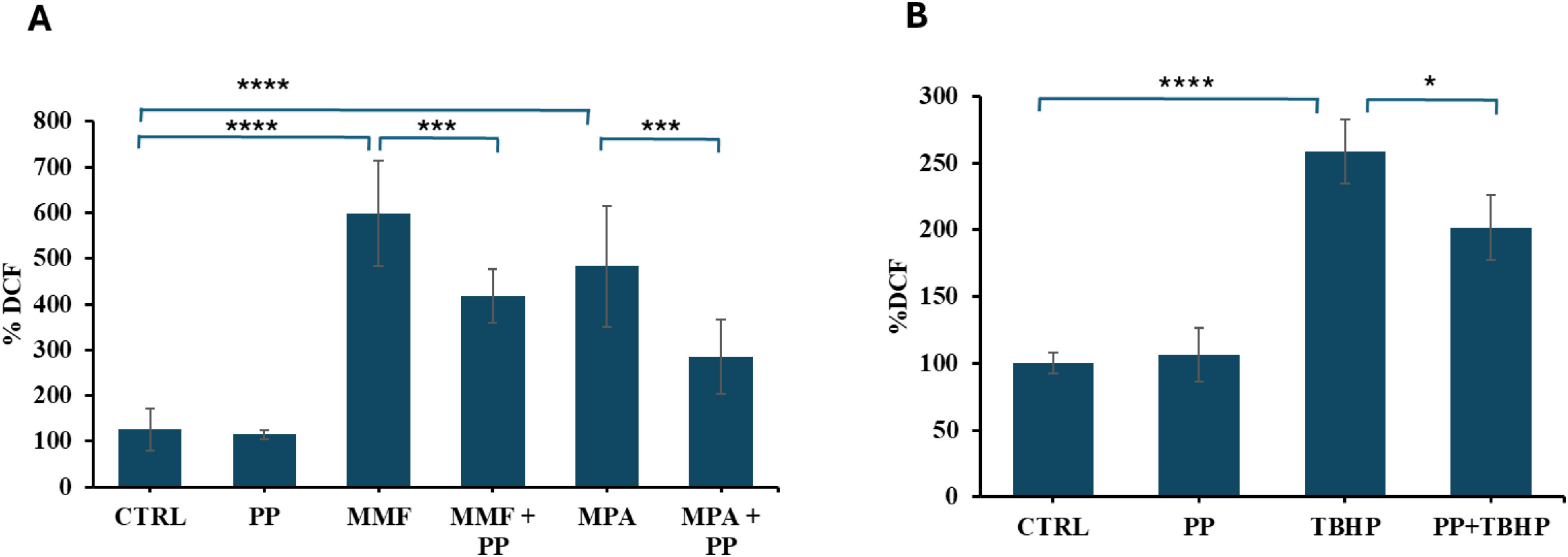
Effect of probiotic-conditioned (PP) on reactive oxygen species (ROS) generation in differentiated CaCo-2 cells. In panel **(A)** cells were exposed for 2 h to mycophenolate mofetil (MMF) or mycophenolic acid (MPA) (both at a concentration of 50 μM) or their vehicle, either in normal cell culture medium or PP. In panel **(B)** cells were incubated in normal medium or PP for 2 h before tBHP (300 μM) was added. Intracellular ROS were measured using the dichlorofluorescein-diacetate (DCF-DA) assay after a 1 h incubation with these treatments. The bar graph shows DCF fluorescence as a percentage of control values ± SD. Asterisks indicate statistical significance after the ANOVA (multiple comparison) analysis: *p < 0.05; ***p < 0.001; ****p < 0.0001.

Since it has been reported that, both the cytotoxicity and the decrease of TJ protein expression induced by MPA are dependent on the increase in intracellular free oxygen radicals (16) we investigated whether PP could protect CaCo-2 cells from these toxic mediators. First, as reported in Figure 4A we confirmed that MMF and MPA, in differentiated CaCo-2 cells, significantly increases intracellular ROS levels, measured as the increment of the fluorescence emitted by DCF as described in the methods section, while PP exhibits a clear antioxidant effect. To determine whether the protective effect of PP extends to general oxidative stress in the intestinal barrier, cells were exposed to the pro-oxidant compound tBHP either in the presence of PP or in standard culture medium. As illustrated in Figure 4B, the tBHP-induced ROS increase was significantly attenuated in the presence of PP, suggesting that PP can protect Caco-2 cells from oxidative damage.

### 3.4 PP decreases ABC1B1 activity

**FIGURE 5:**
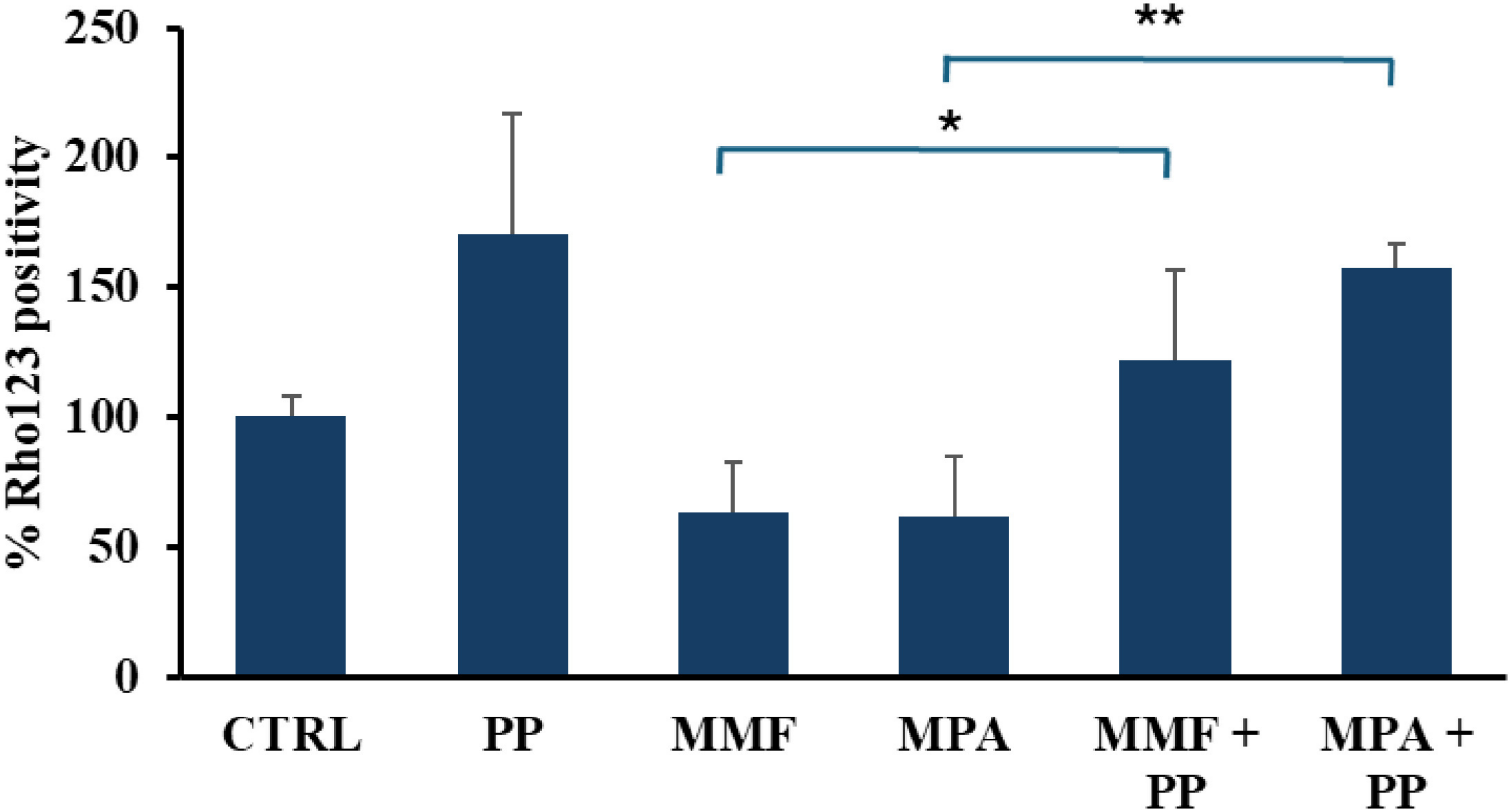
Effect of probiotic-conditioned (PP), mycophenolic acid (MPA), and mycophenolate mofetil (MMF) on ABC1B1 activity. The bar graph shows the mean and standard deviation (SD) of Rhodamine (Rho-123) fluorescence/protein values measured in three experiments with CaCo-2 cells. The cells were incubated for 24 h with either mycophenolic mofetil (MMF) or mycophenolate acid (MPA), both at a concentration of 50 μM, in normal cell culture medium or in PP. Asterisks indicate statistical significance after the ANOVA (multiple comparison) analysis: *p < 0.05; **p < 0.005.

Since probiotics are known to regulate the expression and activity of the drug-extruding pump ABC1B1 (also known as PgP/MDR1) (36), of which MPA is a substrate (37, 38), we investigated whether PP also affects ABC1B1 activity in our experimental system by measuring the intracellular accumulation of its fluorescent substrate, Rho-123. Figure 5 shows the results of these experiments, which revealed an increase in Rho-123 accumulation (and therefore, a decrease in ABC1B1 activity) of about 50% in cells incubated with PP compared to the control group. We also observed that MPA and MMF decreased Rho-123 accumulation, but when cells were exposed to these drugs in the presence of PP, pump activation was completely reversed, resulting in significantly higher Rho-123 accumulation.

**FIGURE 6:**
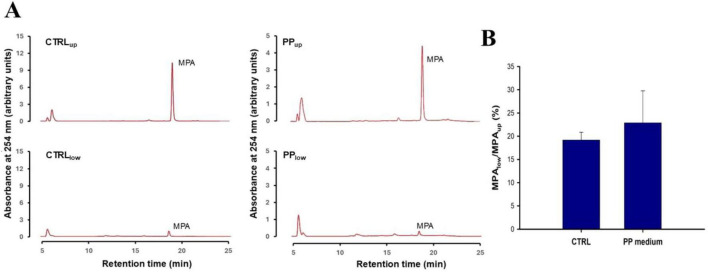
Transport of mycophenolic acid (MPA) across CaCo-2 transwells in the presence of probiotic-conditioned (PP) and control medium. **(A)** Shows representative chromatograms obtained from the upper and lower compartment medium extracts of control and PP-exposed transwells. Experiments were performed in triplicate. As detailed in the Section “2 Materials and methods,” MPA (50 μM) was added to the upper compartment of the transwell, either in normal medium (in the control group) or in the PP medium. The transwell was then incubated for 6 h before the medium was collected and processed. In all groups, MPA eluted with a retention time of approximately 18.5 min. **(B)** Shows bar graph of the mean ± SD of the ratios of the MPA peak areas in the upper and lower compartments of the control and PP transwells. No statistically significant difference between control and PP medium transwells was observed at the *p* < 0.05 significance level using an unpaired Student’s *t*-test.

To assess whether PP modulates ABCB1 expression, we analyzed protein levels by immunoblotting. The results showed that ABCB1 protein levels were not significantly altered following treatment (Supplementary Figure 4), suggesting that the observed differences in Rho-123 accumulation are more likely due to functional modulation of transporter activity rather than changes in total protein expression. Collectively, the experiments with Rho-123 suggest that PP reduces the activity of ABC1B1 in cultured CaCo-2 cells, consequently enhancing the intracellular concentration and decreasing the extracellular efflux of its substrates. To investigate whether this affects the transfer of MPA or MMF across the IB, these drugs were added to the upper compartment of CaCo-2 cell transwells in either PP or control medium. The accumulation of MPA or MMF in the two transwell compartments after a 6 h incubation period was assessed by RP-HPLC analysis. Both in control and PP transwells, the area under the MPA peak in the lower compartment was less than 20% of the area in the upper compartment. This indicates that MPA was predominantly confined to the upper compartment (Figure 6A). To evaluate whether PP modified the ability of MPA to cross the transwell *in vitro*, we calculated the ratio of MPA peak areas in the lower and upper transwell compartments, in both control and PP transwells. As shown in Figure 6B, no significant difference was observed, suggesting that PP-induced inhibition of ABC1B1 pumps did not affect MPA absorption across the transwell.

**FIGURE 7:**
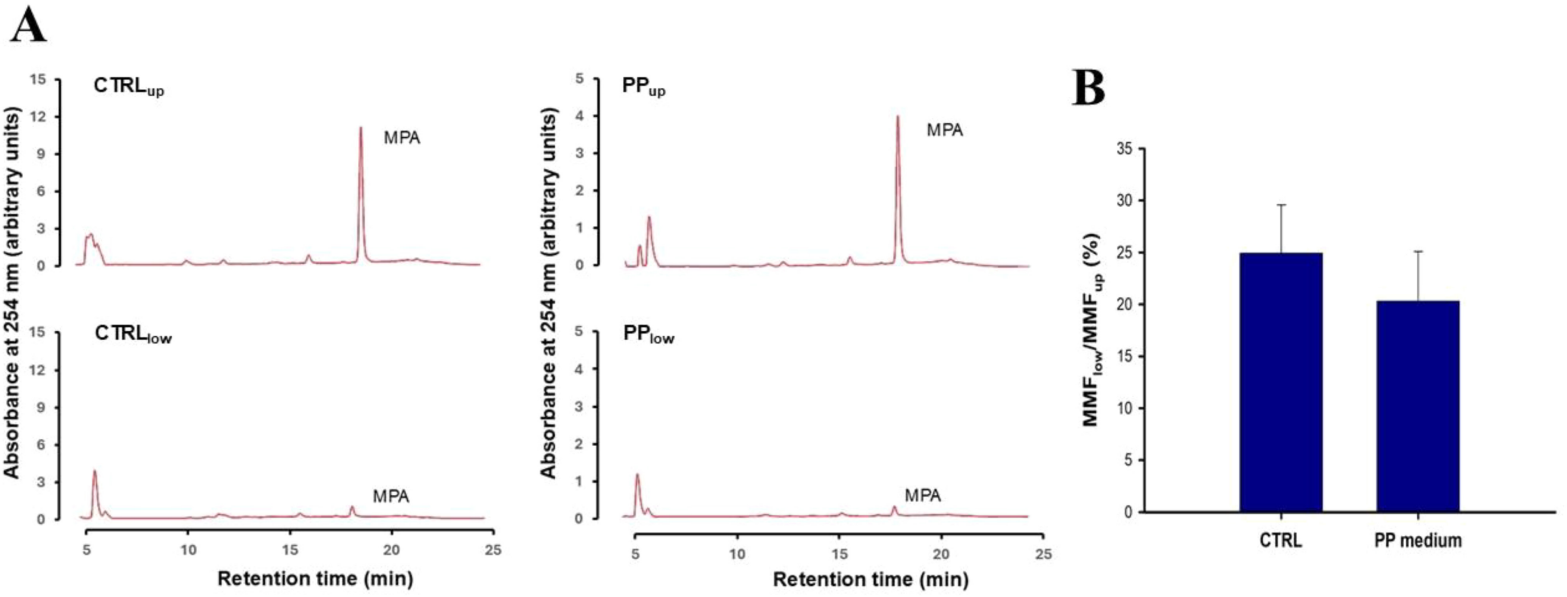
Mycophenolate mofetil (MMF) transport across CaCo-2 transwells in the presence of probiotic-conditioned (PP) and control medium. **(A)** Shows representative chromatograms of the upper and lower compartment medium extracts from the control and PP-exposed transwells. Experiments were performed in triplicate. As detailed in the “Section 2 Materials and methods,” 50 μM of MMF was added to the upper compartment of the transwell, either in normal medium (in the control group) or in the PP medium. The transwell was then incubated for 6 h before the medium was collected and processed. Note that no significant peak was observable at the expected MMF retention time (approximately 10 min with the MMF standard), but distinct peaks were detected at approximately 18.5 min, which is the MPA retention time. **(B)** Shows a bar graph of the mean ± SD of the ratios of the areas of these MPA peaks in the upper and lower compartments of the control and PP transwells. No statistically significant difference between control and PP medium transwells was observed at the *p* < 0.05 significance level using an unpaired Student’s *t*-test.

MMF was extensively converted into MPA when incubated with either PP or the control transwells. In fact, RP-HPLC analysis of the upper compartment medium extracts did not reveal significant peaks with a retention time consistent with unmodified MMF (approximately 10 min in the control experiments), but large peaks consistent with MPA were observed (Figure 7A). These were the only peaks that were quantified. MMF-derived MPA was also found in the lower compartment medium, but in much smaller amounts. The ratio of the areas of the MPA peaks in the lower and upper compartments was approximately 20%. There was no significant difference in the values of these ratios between transwells containing PP or the control medium, suggesting that the transwell IB crossing of MMF-derived MPA was not significantly affected by PP-induced ABC1B1 inhibition (Figure 7B).

## 4 Discussion

In the present study, we investigated the effects of a multistrain probiotic formulation on intestinal barrier integrity and its protective role against MPA- and MMF-induced cytotoxicity.

A fundamental premise of our study was to investigate the early protective actions exerted by the probiotic-conditioned medium (PP) on the intestinal barrier before overt damage induced by MPA or MMF becomes evident. The detrimental effects of these drugs on Caco-2 monolayers, such as decreased viability, loss of TJ proteins, increased ROS production, and impaired barrier function are widely documented in the literature, typically emerging after prolonged exposures of 24–48 h or more. In contrast, at shorter exposure times, MPA/MMF generally induce only modest alterations in barrier integrity. For this reason, our experimental design intentionally focused on early time points (6 h), which represent a critical window to evaluate whether PP can *precondition* intestinal epithelial cells and mitigate the initial molecular disturbances triggered by these drugs. This rationale shaped the entire study: our purpose was not to re-establish the well-known toxicity profile of MPA and MMF, but rather to determine whether PP can enhance barrier resilience at an early stage, before substantial damage occurs. Accordingly, the effects we observed, such as increased TEER, reduced ROS accumulation, and selective upregulation of rapidly responsive TJ proteins (ZO-1 and claudin-5), are fully consistent with this preventive framework, supporting the notion that PP can exert early protective actions on the intestinal epithelium.

MPA and MMF are immunosuppressant drugs widely employed in transplant medicine to prevent organ rejection. Their use is, however, frequently accompanied by diarrhea which may induce IB damage either indirectly, by causing dysbiosis, or directly by enterocyte cytotoxicity (39). The main finding of our study was that the tested preparation increase the tightness of IB and alleviated the cytotoxicity of these drugs. PP’s protective effect on barrier integrity and function likely reflects multiple mechanisms, including early modulation of oxidative stress and TJ function, prior to the onset of drug-induced cytotoxicity. This protective effect was observed in a transwell *in vitro* IB model in the absence of any dysbiotic microbiota. A previous study already demonstrated that probiotic microorganisms may reduce MPA toxicity for the IB but this study was performed in mice and therefore direct and indirect effects could not be dissected (16). Moreover, significant dysbiotic changes were observed in mice receiving MPA, which were attenuated by probiotics pinpointing to an indirect mechanism. Not only, in our study, the probiotic protective effect was observed in the absence of the microbiota and, therefore, it was dysbiosis-independent, but it was also independent from the presence in the system of the probiotic microorganisms themselves. In fact, what we added to the intestinal cells *in vitro* was an acellular conditioned medium prepared by growing the probiotic microorganisms in cell culture medium and, then, removing them by centrifugation and filtration. Therefore, what we observed, strictly speaking, was a postbiotic effect.

MPA, inhibitor of inosine monophosphate dehydrogenase, may cause cell death in intestinal cells by activating the mitochondrial apoptotic pathway. Structural damage to these organelles, a decrease in the mitochondrial membrane potential (ΔΦ_*m*_) and an increase in free radicals have been observed, indeed, in cells exposed to MPA (16). The increase in free radicals caused by mitochondrial damage may worsen cell damage further. In addition, it could explain the decrease in TJ protein expression that has been observed in previous studies in MPA-treated CaCo-2 cells (15, 40, 41). In fact, they induce TJ protein oxidation, which leads to ubiquitination and proteasomal degradation. Additionally, ROS may reduce the expression of genes encoding TJs by directly impacting intracellular transduction pathways that regulate this process, such as the MAPK pathway or NF-κB, or indirectly by causing inflammation and cytokine release. Importantly, decreased TJ expression not only increases IB permeability but also exacerbates cell damage. Indeed, it has been demonstrated that the loss of TJ proteins may act as a death signal itself (42). Unlike in previous studies (26, 40, 41), we did not observe a decrease in tight junction (TJ) protein expression in CaCo-2 cells exposed to MPA or MMF. Several factors could explain this finding, including differences in exposure time or concentration of the immunosuppressors used. Additionally, not all TJ proteins are equally sensitive to free radical stress, which, on the other hand, may modify TJ protein subcellular localization without affecting total content (43, 44). Importantly, the probiotic conditioned medium induced *per se* an increase in TJ protein expression which was still evident in the presence of MPA and MMF.

Given the role of ROS in drug-induced intestinal barrier damage, it is noteworthy that we observed that both the drugs MMF and MPA, as well as the pro-oxidant compound tBHP, increased ROS production to a lesser extent in cells exposed to probiotic-conditioned medium compared to control cells cultured in normal medium. This finding suggests that the protective effect of the probiotic conditioned medium could depend at least partly on its ability to reduce intracellular ROS. Previous studies showed that probiotics can reduce free radicals through various molecular mechanisms (45, 46). These mechanisms include releasing antioxidant compounds, such as glutathione, enzymes, such as catalase and peroxidase, metal ions chelators, exopolysaccharides and soluble mediators, such as SCFAs (45–50). Interestingly, evidence suggests that probiotics may preserve tight junction (TJ) expression in the presence of free radical stress (51). Collectively, the data here reported show that the well-known ability of probiotics to protect the IB (35) also may applies to MPA/MMF induced damage and could be dependent on a decrease in intracellular ROS levels and to an increase in TJ proteins.

Efflux pumps, such as ABC1B1, are a major protective factor against drug-induced toxicity because they expel toxic compounds from the cytoplasm, thereby keeping intracellular concentrations low. Evidence suggests that MPA is an ABC1B1 substrate because knockout mice for this pump have lower circulating MPA concentrations than wild-type mice (38). Several toxic agents and drugs induce ABC1B1 expression by activating the intracellular receptors PXR and CAR (52). Additionally, the expression of this pump is modulated by free radicals in a concentration- and time-dependent manner, mostly through the transcription factor Nrf2 (53, 54). More specifically, free radicals have been shown to increase ABC1B1 protein expression in CaCo-2 cells (55). Our experiments with Rho-123 revealed that MPA and MMF may increase ABC1B1 activity in CaCo-2 intestinal cells, without significant changes in level of protein expression. A wealth of experimental data shows that probiotics increase ABC1B1 expression and activity in intestinal cells (36, 56, 57), our finding that probiotic-conditioned medium reduced pump activity and reversed the effects of MPA and MMF was unexpected. One possible explanation for our results is that the probiotic-conditioned medium reduced the intracellular concentration of ROS due to its protective effects against the toxicity of these immunosuppressant drugs. As mentioned above, ROS are an important driver of ABC1B1 expression. A decrease in ABCB1B1 pump activity due to probiotic exposure could increase the intracellular concentration of MPA, thereby enhancing its toxicity. However, this effect is likely negligible because it should be offset by an increase in intracellular MPA concentration and subsequent increase in ABCB1B1 activity.

Since MPA is an ABC1B1 substrate (37, 38), another possible consequence of decreased pump activity induced by PP could be increased MPA accumulation in the lower transwell compartment due to decreased flux from the cell cytoplasm to the upper compartment. However, our findings did not support this hypothesis. PP did not modify the ratio of MPA amounts in the lower and upper compartments compared to control transwells. Wang et al. studied ABC1B1 knockout mice and reported evidence of a role for ABC1B1 in MPA pharmacokinetics *in vivo* (38). However, the observed effect was small. They found higher MPA plasma concentrations in knockout mice than in wild-type mice 30 min after oral gavage administration of the drug, but no difference was observed at later time points. They suggested that the high MPA bioavailability could compensate for possible drug loss due to the knockout of the pump (38). It is important to note that our incubation time was 6 h. The behavior of MMF in our *in vitro* system was much more complicated. MMF is a prodrug that must be desterified to convert to the active form, MPA (58). Our HPLC data showed that this conversion occurred in Caco-2 transwells. This finding is consistent with evidence showing that intestinal cells mainly desterify MMF via CES2 and to a lesser extent via CES1 (58). These enzymes are also expressed in Caco-2 cells. However, when we analyzed the effect of PP on the transfer of MMF-generated MPA across the transwell membrane, we obtained results similar to those with exogenous MPA. In this case, too, there was no significant consequence of PP-induced inhibition of pump activity.

Since all experiments in this study used an acellular probiotic conditioned medium free of bacterial cells, the observed effects were likely caused by soluble mediators released during incubation with probiotic microorganisms when preparing the medium. Conversely, we can exclude the possibility that the reduced IB damage was due to a lower concentration of the offending drug in the probiotic preparation compared to the control medium. Although we added the test drugs (MPA or MMF) at the time of probiotic bacterial inoculation during preparation of the probiotic-conditioned medium, no evidence of accumulation or degradation of these compounds by bacterial cells emerged in the HPLC analysis of the conditioned medium. Further studies will be necessary to identify the soluble factors in the probiotic conditioned medium which are responsible for barrier protection. However, some likely candidates can be hypothesized. Among them SCFA are known to increase IB integrity, to induce the expression of TJ and to reduce oxidative stress (9, 59–61). Other secreted factors that could have played a role in the protective effects of the probiotic conditioned medium include enzymes and small molecules with antioxidation activity (see above). An important advantage of our experimental model involving the coincubation of MMF and MPA with probiotic bacteria during the preparation of the conditioned medium is that it allowed the potential interaction between these microorganisms and drugs. It is now well-established that bacteria may sense the presence of drugs also including non-antibacterial compounds and respond by releasing soluble mediators and extracellular vesicles (62, 63). It will be interesting to explore in future studies whether any of the substances protecting the IB from MFA toxicity are actually induced or released in the presence of this drug or of its ester MMF.

The results of our study could have important practical implications. They expand, indeed, the list of possible applications of the protective effect of probiotics for the IB to the specific setting of MPA toxicity. Probiotics are known to enhance epithelial barrier integrity, upregulate tight junction (TJ) proteins, and mitigate oxidative and inflammatory stress in various conditions (35, 64–66). However, probiotics are not routinely used to prevent or counteract the intestinal toxicity of MPA and MMF. In fact, this toxicity is typically treated by discontinuing the medication and switching to a different immunosuppressant. Our study’s results support the use of probiotics to maintain MPA or MMF therapy in patients who initially experience gastrointestinal toxicity from these drugs. Our study has an important limitation in that it was performed *in vitro*, so it must be corroborated further *in vivo* studies, possibly in humans. One important finding from our experiments is that probiotic bacteria are not necessary for protection, as this can be fully exerted by an acellular preparation from their cultures. This postbiotic effect could be advantageous from a clinical standpoint, especially for transplant patients, as probiotics have occasionally been associated with opportunistic infections in immunocompromised patients (67).

In conclusion, we showed that factors released by probiotic bacteria into their culture medium may reduce the damage caused to the IB by MPA and MMF by a mechanism that involves an increase in cell viability and TJ protein expression possibly dependent on the reduction of intracellular ROS concentration.

## Data Availability

The original contributions presented in this study are included in the article/Supplementary materials, further inquiries can be directed to the corresponding author.
