# Supplementary material for: Enhancement of intestinal barrier function and alleviation of mycophenolic acid toxicity by a probiotic-conditioned medium in vitro
Source: Front Nutr. 2026 Apr 14;13:1809197. doi: 10.3389/fnut.2026.1809197 (PMC13121142; doi:10.3389/fnut.2026.1809197)
Supplement: Supplementary file 1 [file Supplementary_file_1.pdf]

Probiotic Bacteria Enhance Intestinal Barrier Function and Alleviate Mycophenolic Acid Toxicity in an In Vitro Model

SUPPLEMENTARY MATERIAL

1.MPA and MMF do not exert bacteriostatic or bactericidal effects on probiotic cultures

The effect of mycophenolic acid (MPA) and mycophenolate mofetil (MMF) on the growth and survival of the probiotic preparation was evaluated using viable plate counts on appropriate culture media (17). The microbial load was measured at three time points: immediately after inoculation (T0) and after three (T3) and six (T6) hours of incubation at 37 °C in DMEM. The results of these experiments are shown in Supplementary Figure 1.

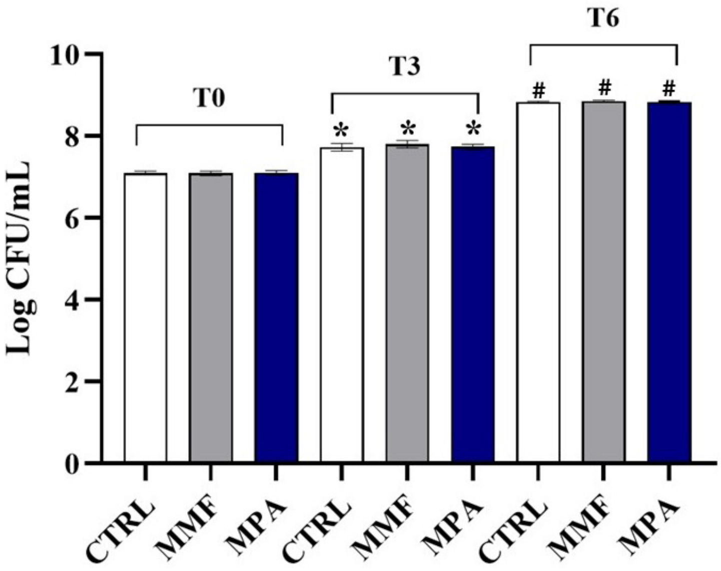

Supplementary Figure 1. Growth of probiotic bacteria in the presence of MPA or MMF. The bar graph reports the mean ± SD of microbial load values (Log CFU/mL) measured in three independent experiments at the time of inoculation (T0) and after 3 (T3) and 6 h (T6) of incubation at 37 °C in the presence of MPA or MMF (both at a concentration of 50 µM) or their vehicle (CTRL). Data were analyzed using GraphPad Prism version 9.0 (GraphPad Software Inc., California, USA). Statistical differences among groups were assessed by one-way analysis of variance (ANOVA), followed by Dunnett's multiple-comparison test. *= $p<0.05$ vs T0, #= $p<0.05$ vs T3 and T0.

At T0, all experimental conditions (CTRL, MMF, and MPA) showed comparable microbial loads, with values of approximately 7 log CFU/mL. After 3 h of incubation, microbial counts increased to 7.81 ± 0.03 log CFU/mL in the presence of MMF and to 7.73 ± 0.07 log CFU/mL in the presence of MPA. A further increase was observed after 6 h, reaching 8.86 ± 0.02 log CFU/mL for MMF-treated samples and 8.82 ± 0.02 log CFU/mL for MPA-treated samples. No statistically significant differences (p >

0.05) were detected between drug-treated samples and the control condition at any of the analysed time points, indicating that neither MPA nor MMF exerts bacteriostatic or bactericidal effect on the tested probiotic preparation.

2. MPA and MMF concentrations are stable during probiotic culture growth

To investigate whether the microorganisms of the probiotic preparation could degrade MPA or MMF, we analyzed, by RP-HPLC, extracts of their cultures in Caco-2 cell medium supplemented with either of these drugs or their vehicles, taken immediately after inoculation and after three and six hours of incubation. Examining the recorded chromatograms revealed no extra-peaks suggestive of significant degradation of MPA or MMF at any of the examined times (data not shown). Furthermore, we did not observe any difference in the extracellular concentration of the immunosuppressant drug at the three different time points, for either MPA or MMF (Supp. Figure 2A and B, respectively). Similarly, we observed no differences between the bacterial cultures and their controls containing vehicle only at any time point (Figure 2A and B). These data collectively show that there is no significant change in MPA or MMF concentration during incubation with probiotic microorganisms for PP preparation.

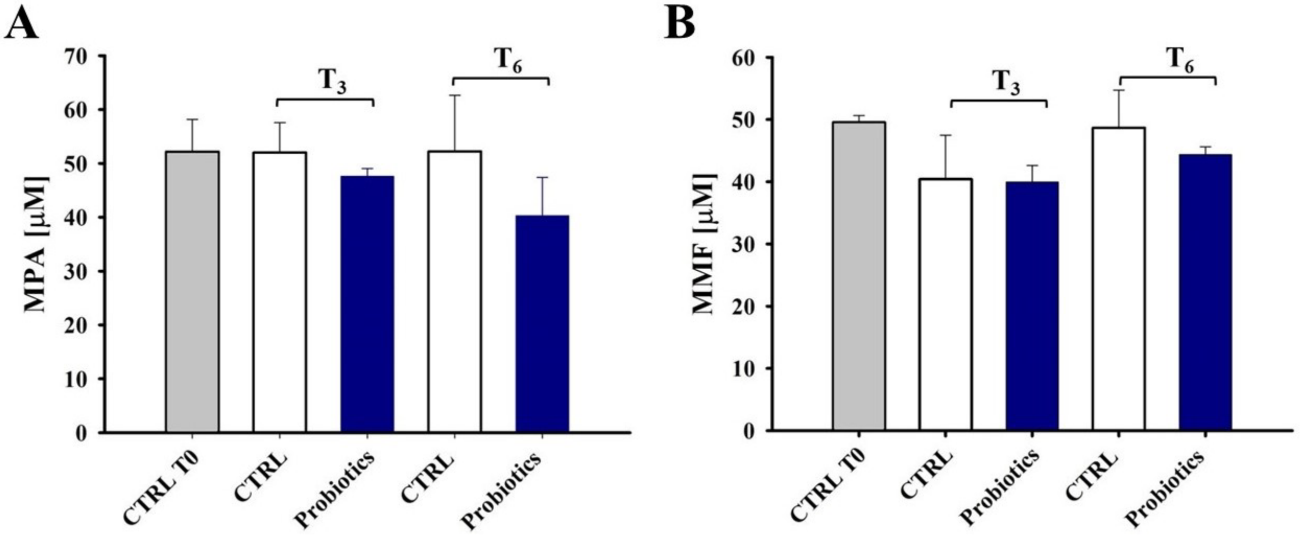

Supplementary Figure 2. Concentration of MPA and MMF in the probiotic culture medium. The bar graph reports the mean ± SD of the concentrations of MPA (panel A) and MMF (panel B) measured in the probiotic culture medium in five independent experiments at the time of inoculation (T0) and after 3 (T3) and 6 h (T6) of incubation at 37 °C. Drug concentration was determined by RP-HPLC in culture medium samples processed as described in the methods section. For quantification, hydrochlotiazide was used as an internal standard and standard MPA and MMF were used for calibration. Statistical differences among groups were assessed by ANOVA, followed by Bonferroni-corrected post hoc comparisons. The threshold for statistical significance was set at $p<0.01$.

3. The protein level of Occludin and Claudin-1 did not significantly change after treatments with PP, MPA and MMF

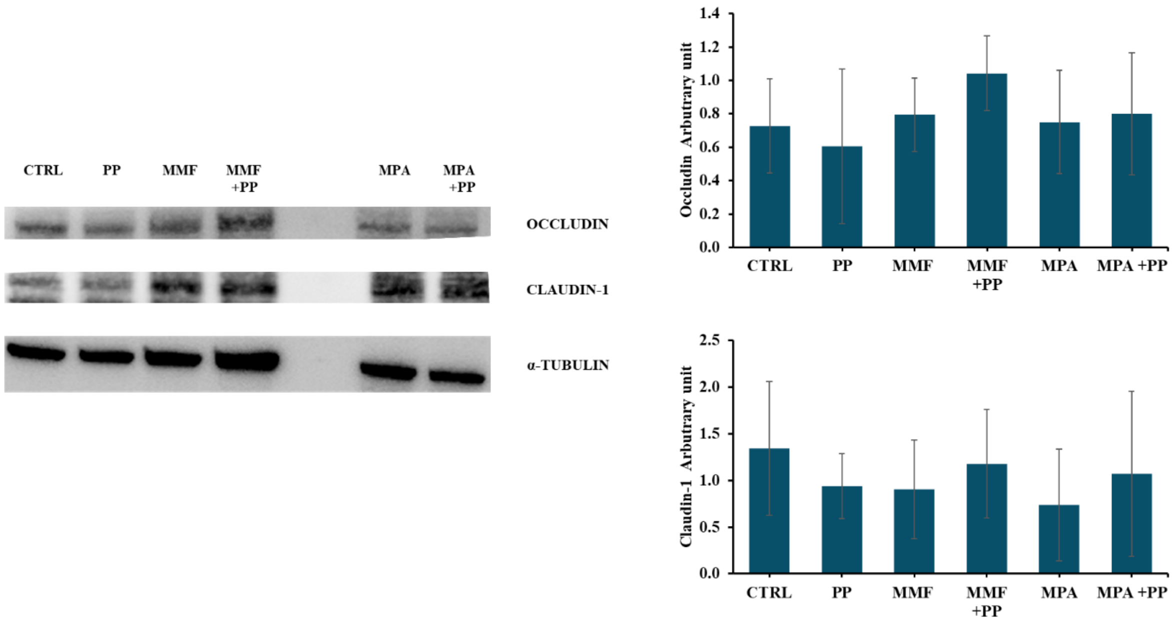

Figure S3. Effect of PP, MPA and MMF on the expression of Occludin and Claudin-1 protein. The immunoblots show the expression levels of Occludin and Claudin-1 proteins in lysates of differentiated CaCo-2 cells that were exposed for 6 hours to MMF or MPA (both at a concentration of 50 µM) or their vehicle, either in normal cell culture medium or PP. The bar graph shows the mean and standard deviation (SD) of the values of densitometric analysis (arbitrary unit) obtained in three experiments.

4. The protein level of ABC1B1 did not significantly change after treatments with PP, MPA and MMF

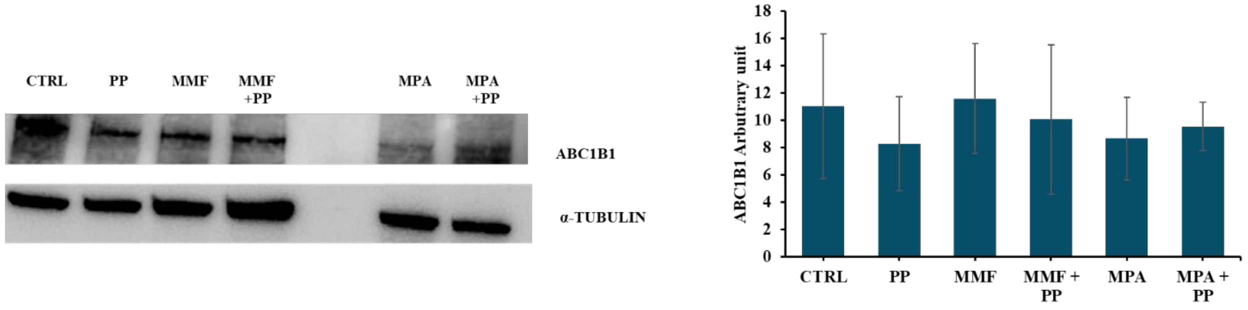

Figure S4. Effect of PP, MPA and MMF on the expression of ABC1B1 protein. The immunoblot show the expression levels of ABC1B1 protein in lysates of differentiated CaCo-2 cells that were exposed for 24 hours to MMF or MPA (both at a concentration of 50 μM) or their vehicle, either in normal cell culture medium or PP. The bar graph shows the mean and standard deviation (SD) of the values of densitometric analysis (arbitrary unit) obtained in three experiments.
